# Glutathione peroxidase 4 overexpression induces anomalous subdiffusion and impairs glioblastoma cell growth

**DOI:** 10.1186/s13036-024-00472-x

**Published:** 2024-12-21

**Authors:** Nahom Teferi, Akalanka Ekanayake, Stephenson B. Owusu, Thomas O. Moninger, Jann N. Sarkaria, Alexei V. Tivanski, Michael S. Petronek

**Affiliations:** 1 Department of Neurosurgery, University of Iowa, Iowa City, IA USA; 2 Department of Chemistry, University of Iowa, Iowa City, IA USA; 3 Department of Radiation Oncology, University of Iowa, Iowa City, IA USA; 4 Central Microscopy Research Facility, Carver College of Medicine, University of Iowa, Iowa City, IA USA; 5 Department of Radiation Oncology, Mayo Clinic, Rochester, MN USA

**Keywords:** Ferroptosis, Glioblastoma, Cell stiffness, Cell motility, Tumor progression, Glutathione peroxidase 4

## Abstract

Glioblastoma tumors are the most common and aggressive adult central nervous system malignancy. Nearly all patients experience disease progression, which significantly contributes to disease mortality. Recently, it has been suggested that recurrent tumors may be characterized by a ferroptosis-prone phenotype with a significant decrease in glutathione peroxidase 4 (GPx4) expression. This led to the hypothesis that GPx4 expression negatively influences GBM cell growth. This study utilizes a doxycycline inducible GPx4 overexpression model to test this hypothesis. Consistently, the overexpression of GPx4 significantly impairs cell growth and colony formation while also causing an accumulation of cells in G1/G0 phase of the cell cycle. From a biophysical perspective, GPx4 overexpressing cells have significantly greater surface area, increased Young’s modulus, and experience anomalous sub-diffusion as opposed to normal diffusion associated with Brownian motion. Moreover, analysis of patient derived GBM cells reveal that cell growth rates, plating efficiency, and Young’s modulus are all inversely proportional to GPx4 expression. Therefore, GPx4 appears to be a biophysical regulator of GBM cell growth that warrants further mechanistic investigation in its role in GBM progression.

**Supplementary Information:**

The online version contains supplementary material available at 10.1186/s13036-024-00472-x.

## Introduction

Glioblastoma (GBM) remains the most common and aggressive primary central nervous system tumor type with a median age at diagnosis of 66 years. It accounts for 14.2% of all CNS tumors and 50.9% of all malignant tumors in the CNS with an annual incidence of 3.27 per 100,000 in the US population [1]. Despite an aggressive standard of care treatment regimen of concurrent and adjuvant temozolomide and radiation therapy, patient outcomes remain poor with a median overall survival of 14.6 months, 2-year survival rate of 26.5%, 5-year survival rate of 9.8% and progression-free survival of 6–7 months [1–3]. Recent therapeutic advancements including tumor-treating fields and utilization of pharmacological ascorbate have shown promise towards improved overall survival of 18.8 and 19.6 months, respectively [4, 5]. Despite the promise of these newer therapeutic approaches, nearly all GBM patients experience disease progression. Following disease progression, there is an even poorer prognosis with a median progression-free survival of 1.5-6 months and a median overall survival of 2–9 months [6–8]. Thus, there remains a dire need for elucidating the molecular mechanisms involved in GBM progression to advance the management of recurrent and progressive GBM.

Currently, the underlying mechanism(s) of GBM progression remain unclear. A recent retrospective report has shown that there are significant, dynamic changes in ferroptosis-related protein expression in primary and recurrent GBM [9]. Immunohistochemical evaluation revealed a mild increase in pro-ferroptotic enzymes like acyl-CoA-synthetase 4 (ACSL4) and a significant, 3-fold decrease in glutathione peroxidase-4 (GPx4) in recurrent GBM tumors relative to the corresponding primary tumor. These data indicate that there is a metabolic evolution that occurs during disease progression that is pro-ferroptotic in nature and may support tumor growth. Consistently, depletion of ACSL4 and GPx4 overexpression has been shown to diminish GBM tumor necrosis and aggressiveness in pre-clinical models of GBM [10]. Thus, it can be hypothesized that a ferroptosis-prone phenotype can aid in GBM progression.

GPx4 is a central, negative regulator of ferroptosis due to its ability to remove phospholipid hydroperoxides [11]. However, the exact mechanism(s) of how altered GPx4 expression and the emergence of a pro-ferroptotic phenotype can alter tumor aggressiveness and modulate GBM recurrence are uncertain. Therefore, a better understanding of the mechanistic impacts of GPx4 on GBM cell biology will provide considerable insights into the process of GBM progression. The overarching goal of this study is to mechanistically evaluate the role of GPx4 in GBM cells at the biophysical level using a doxycycline-inducible model of GPx4 overexpression to test the hypothesis that GPx4 negatively regulates GBM cell growth.

## Materials and methods

### Cell culture

All glioma cells (U118, ATCC HTB-15; U251 Millipore Sigma, 09063001) were cultured in DMEM-F12 media (15% FBS, 1% penicillin-strep, 1% Na-pyruvate, 1.5% HEPES, 0.1% insulin, and 0.02% fibroblast growth factor) and grown to 70–80% confluence at 21% O_2_. Patient-derived glioblastoma cells (GBM06/GBM39 are primary GBM cells from a male donor, GBM76 is a recurrent GBM from a male donor) were a gift from Dr. Jann Sarkaria, MD (Mayo Clinic, Rochester, MN). All cells were confirmed to be mycoplasma negative by the University of Iowa Genomics Core before use. Commercial cells were used for up to 15 passages and patient-derived cells were used for up to 10 passages.

The GPx4+-pTRIPZ vectors were provided by the laboratory of Douglas Spitz and used as previously described [12]. To produce lentivirus, TSA201 cells were used along with VSV-G and psPAX2 helper vectors (Addgene). Virus was collected from TSA201 cell cultures, centrifuged to remove cell debris, and filtered using 0.45 μm filters from the ZymoPURE^tm^ II Plasmid Midiprep Kit (Zymo Research, Irvine CA, USA). Cells were plated and allowed to grow for 24 h, and then virus was added to cells with 8 µg/mL of polybrene for a total of 48 h, with fresh virus being added after 24 h. Following transduction, cells were selected with 2.5 µg/mL puromycin. The general population that survived the puromycin selection where then validated for overexpression by treating them with 1 µg mL^− 1^ doxycycline hyclate (Fisher Bioreagents BP2653-5, Geel, Belgium) for 48 h. Low density cell suspensions were then grown in to 96 well plate to form single cell clones. Picked clones were also treated with 1 µg mL^− 1^ doxycycline for 48 h to validate the overexpression.

### Atomic force microscopy

Cells were treated for the designated time period, trypsinized, and counted. Cells were then plated as single cells (≈ 8000 cells) on a cover slip in a 60 mm^3^ dish 24 h prior to analysis. All cell stiffness measurements were performed using a Molecular Force Probe 3D AFM (Asylum Research, Santa Barbara, CA) nanoindentation measurements using AFM tips (Nanotools, Germany, biosphere™ B2000-CONT) with a high-density, diamond-like carbon sphere of radius 2 μm attached at the end of a flexible cantilever with a spring constant of 0.2 N/m. Single cells were located using an AFM optical camera before the nanoindentation measurements. Nanoindentation measurements involved collecting force–vertical displacement curves in contact mode in a phosphate-buffered saline buffer at 22 ± 2 °C at an approximate center of each cell with a maximum applied loading force of 3 nN and a 500 nm/s approach velocity. At least three repeated force-vertical displacement measurements were collected per each cell and typically ten individual cells were measured per each sample. The force-vertical displacement profiles were converted to force-indentation distance and the approach to the surface data was fit using the Hertzian elastic contact model to calculate the corresponding cell stiffness (Young’s modulus) as described previously [13]. For the model, the AFM tip was modeled as a sphere with a radius of 2 μm, and Poisson ratios of the tip and cell were assumed to be 0.2 and 0.25, respectively. For each cell, fitted Young’s modulus results based on at least three repeated measurements were averaged to yield an average cell stiffness value.

### Cell growth

To evaluate cell growth rates, 100,000 cells for each group was plated on day 0. Cells were treated daily with 1 µg mL^− 1^ doxycycline in fresh media (total load of 6 µg mL^− 1^ doxycycline over 6 days) and counted to evaluate cell growth. Cells were harvested with trypsin and the total attached cell population was counted using a Beckman Coulter counter. The number of cells at each time point were normalized to the control time point (100,000) to evaluate relative growth.

### Colony formation

Cells were treated, washed, and trypsinized. Following trypsinization, cells were counted and plated as single cells in a 6-well dish (≈ 500–1000 cells per well). Cells were left undisturbed for 7–10 days to allow for colony formation. Colonies were then washed with 70% EtOH for fixation and stained with Coomassie blue. Stained colonies (≥ 50 cells) were counted under a microscope.

### Cell migration

Following 24–48 h of incubation, 600,000 cells were plated in the top well of a trans-well chamber (8 μm pore size) in 500 ul of 1% FBS. The trans-well chamber was suspended in 1 ml of DMEM/F12 BR15 media in a 24-well dish for 24 h. Following incubation the media was removed from the top of the trans-well and the remaining cells on top of the membrane were removed using a cotton swab. Next, the trans-well chamber was removed from the 6-well plate and the migrating cells on the bottom of the membrane were stained by placing the trans-well chamber into 1 mL (concentration = 0.5 mg/mL) of a Thiazolyl Blue Tetrazolium Bromide (MTT) solution for 1-hour. Following incubation in MTT, the stained trans-well chamber membrane was incubated at 37 °C for 20 min in 300 µL of DMSO. Finally, the stained DMSO solution was transferred to a 96-well plate, and the absorption at 550 nm was measured using a plate reader to evaluate the migrating cells. The absorbance of the treated cells was normalized to the control cells to approximate the relative cell migration.

### Cell cycle analysis

Prior to cell cycle analysis, cells were trypsinized, centrifuged, and the pellets were fixed in 70% EtOH and stored at 4 °C. The fixed cells were stained with 1 µg mL − 1 propidium iodide (PI) (catalog #P4170-25MG, Sigma-Aldrich) and the cell cycle distribution was analyzed with a UV-LSR flow cytometer, by measuring the red fluorescence of the PI-stained DNA content. Cell cycle distribution (%) based on DNA content was calculated using FlowJo V10 software.

### Live cell tracking

Cells were treated for the designated time period, trypsinized, and counted. Cells were then plated as single cells (≈ 8000 cells) in a glass-bottom 6-well plate 24 h prior to analysis. Prior to analysis cells were stained for 3 h with NucSpot^®^ 488 Live Cell stain per manufacturer’s instructions (Biotium #40081, Freemont, CA). Live cells were imaged with a Zeiss LSM 980 AiryScan2 confocal microscope temporally in 15-minute intervals to evaluate cell motility. Image analysis was performed using Oxford Imaris to calculate mean squared displacement.

### Western blotting

Cells were lysed in 1X RIPA (Sigma-Aldrich) and total protein was quantified using DC™ protein assay kit (Bio-Rad). 20 µg of total protein from cell lysates was used for western blotting. Electrophoresis was carried out at 100 V on a 4–20% pre-cast gradient gel (Bio-Rad) for 60 min. PVDF membrane (Bio-Rad) was used to transfer proteins at 4° C, 100 V for 60 min. Following transfer, the membrane was blocked using 5% non-fat dry milk in 0.2% PBS-Tween (PBST) for 2 h at room temperature. At 4 °C, primary antibody incubation was done overnight. Gpx4+ (1:1000, Abcam) and β-actin (1:1000, Cell Signaling) primary antibodies were used. The membrane was then washed 3 times for 10 min with PBST and incubated with horseradish peroxidase-conjugated anti-mouse secondary antibody (1:10,000–1:20,000; Cell Signaling) for 1 h at room temperature. Following 3 × 10-minute PBST washes, a chemiluminescent kit (Super Signal West Pico & Super Signal West Femto, Thermo Scientific) was added to the membrane and exposed on an X-ray film (Research Products International).

## Results

**Fig. 1:**
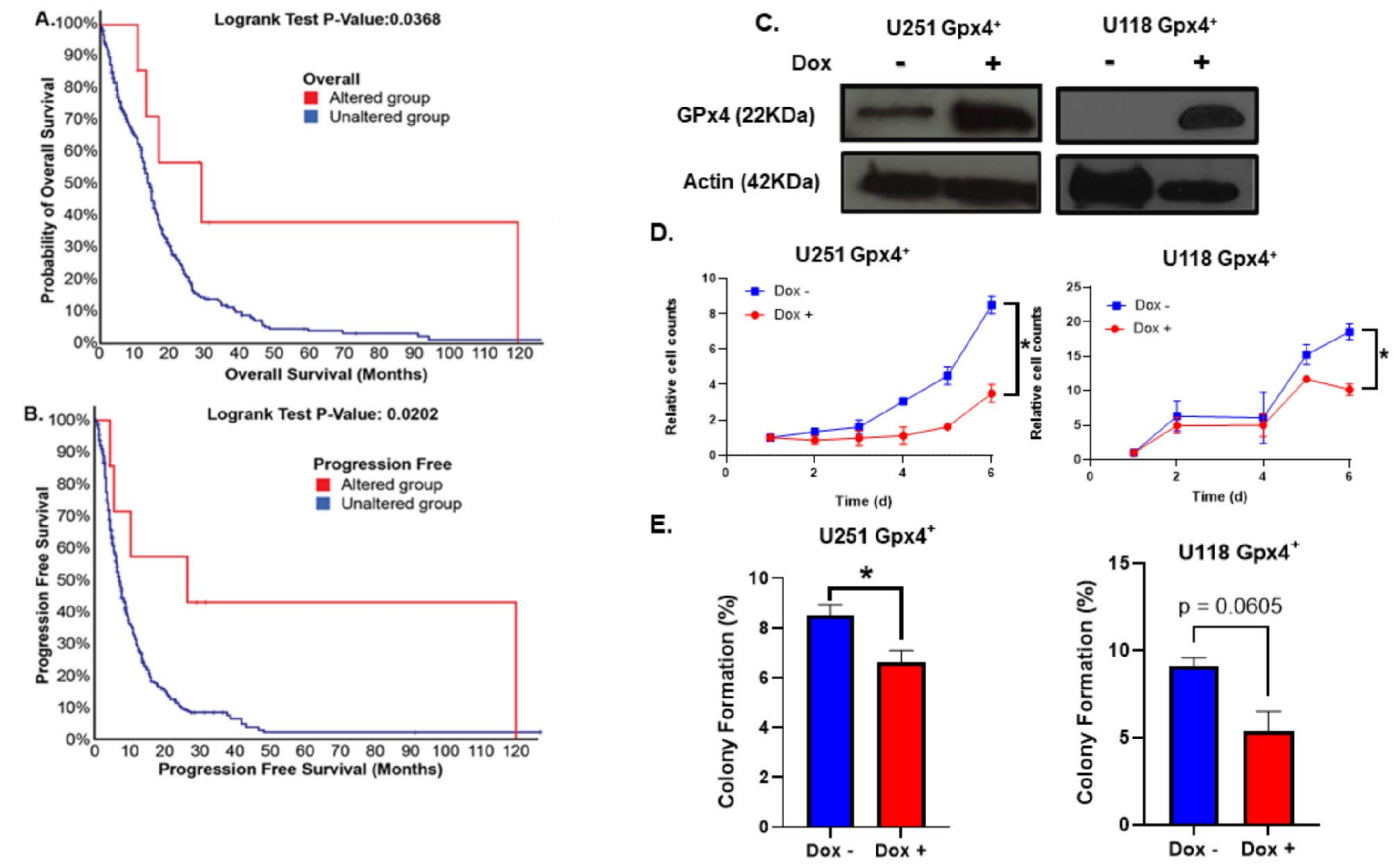
GPx4 overexpression impairs GBM cell growth. **A/B**: From The Cancer Genome Atlas PanCancer Atlas (cbioportal.org, firehouse legacy database, accessed on 7-11-2023), 7 out of 378 (1.9%) samples (Altered group, red) had GPx4 mutations with 5 out of 7 mutations observed being amplifications of GPx4. These mutations were associated with increased overall survival (**A**) and progression-free survival (**B**). **C**: Western blot showing confirmation of overexpression of Gpx4 in U251 and U118 clonal lines following 24 h treatment with 1 µg mL^− 1^ doxycycline. **D**: 100,000 Gpx4 overexpressing U251 and U118 cells were plated on day 0 and then treated daily with 1 µg mL^− 1^ doxycycline in fresh media (total load of 6 µg mL^− 1^ doxycycline over 6 days) and counted to evaluate cell growth. Error bars represent mean ± SD of three independent experiments with *p < 0.05 using a two-way ANOVA test with post-hoc multiple comparisons to compare each time point. **E**: Gpx4 overexpressing U251 and U118 cells were treated daily with 1 µg mL^− 1^ doxycycline for 6 days, counted, and plate as single cells to assess colony formation. Error bars represent mean ± SEM of three independent experiments with *p < 0.05 using a Welch’s T-test

Following the intriguing results showing a pro-ferroptotic phenotype associated with GBM progression, this study aimed to mechanistically evaluate the relationship between GPx4 and GBM growth. First, an analysis of GPx4 expression was conducted using The Cancer Genome Atlas (TCGA). According to the Firehouse legacy database, GPx4 mutations in GBM are rare with only 7 out of 378 (1.9%) samples having GPx4 mutations. Interestingly, 5 out of 7 mutations observed were amplifications of GPx4. Moreover, these mutations were associated with a significant increase in overall survival and progression-free survival (Fig. 1A, B). These results further suggest that GPx4 expression negatively regulate GBM aggressiveness. To further interrogate the role of GPx4 on GBM cell growth, we used a doxycycline-inducible GPx4 overexpression model (Fig. 1C). This was done using a previously established doxycycline-inducible vector that when packaged as a lentivirus has been shown to have minimal effects on cell growth without doxycycline, meanwhile the doxycycline doses used have shown no effects on parental tumor cell growth, which we anticipated to be the case in this study [14]. We first compared the growth rates of the parental U251 cells and U251 Gpx4^+^ cells which revealed that U251 Gpx4^+^ cells do tend to grow slower (doubling times = 17.1 ± 0.3 h and 44.7 ± 0.7 h, respectively) – an effect that is likely the result of the clonal selection process. Importantly, 72 h of daily 1 µg mL^− 1^ doxycycline (3 µg mL^− 1^ total) showed had no significant effect on U251 cell growth as there was no difference in doubling time with respect to the parental U251 cells (doubling time = 17.6 ± 0.3 h) which indicates that any changes in growth in the GPx4^+^ cells can be presumed to be the result of the induction of GPx4. Following this characterization, this doxycycline inducible GPx4 overexpression model was utilized to evaluate the causal effects of GPx4 on GBM growth in vitro. Consistently, it was observed that doxycycline-treated cells (U251 and U118) have a significant decrease in cellular proliferation and colony formation (Fig. 1D, E). Therefore, GPx4 overexpression is capable of impairing GBM tumor growth and cell viability.

**Fig. 2:**
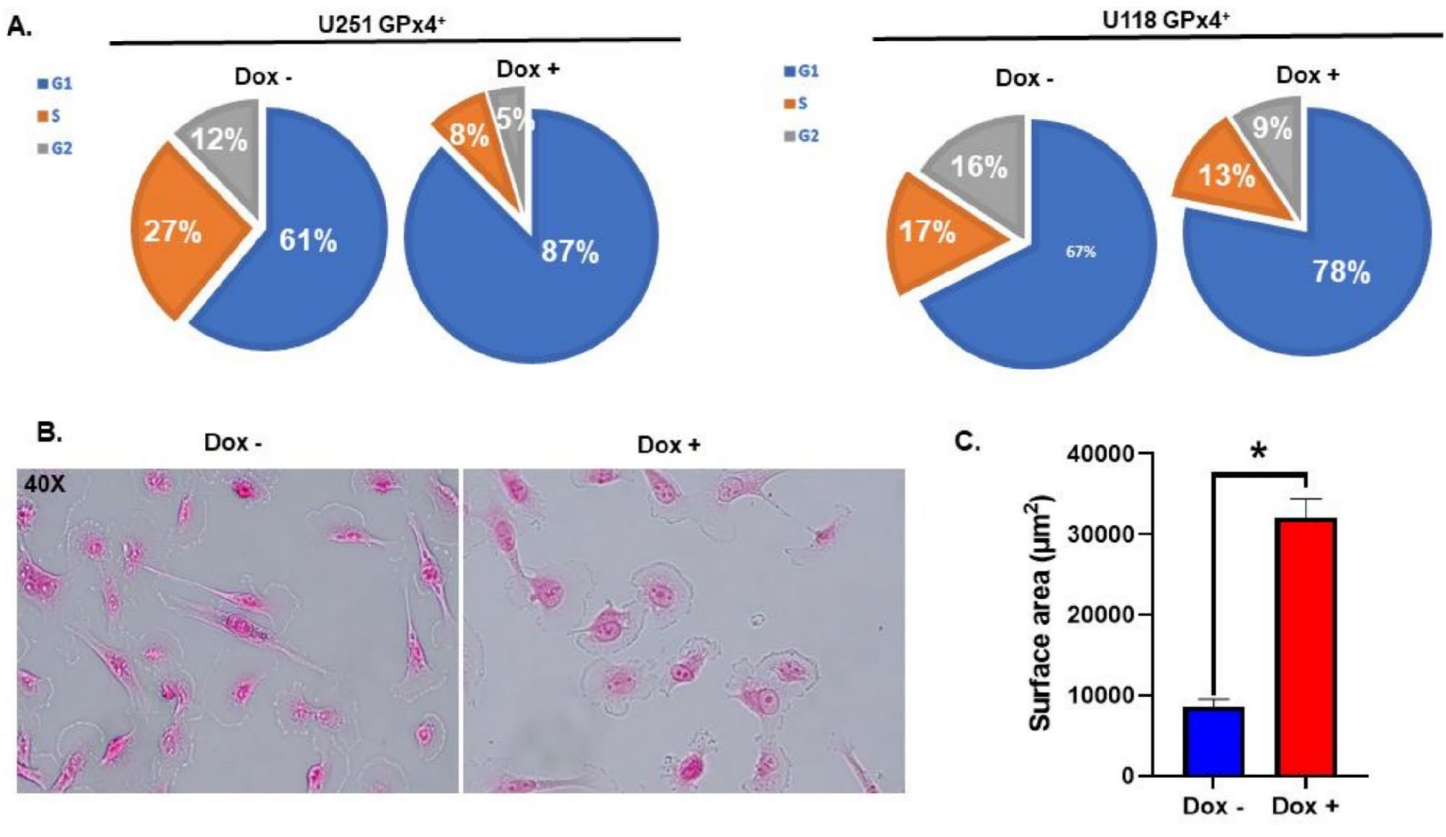
GPx4 overexpression impairs cell cycle progression associated with increased cell size. **A**: Gpx4 overexpressing U251 and U118 cells were treated daily with 1 µg mL^− 1^ doxycycline for 6 days, harvested, and fixed for cell cycle analysis with propidium idodide (1 µg mL^− 1^) using flow cytometry. **B/C**: Gpx4 overexpressing U251 cells were treated with 1 µg mL^− 1^ doxycycline for 6 days, stained with nuclear fast red, and imaged with light microscopy (40X, **B**). Cell surface area was quantified using ImageJ software (**C**). Error bars represent mean ± SEM of *n* = 10 40X fields where *p < 0.05 using a Welch’s T-test

These results led to a more robust interrogation on the effects of GPx4 on GBM cell biology. Consistent with the impairment of cell growth, cell cycle analysis revealed that GPx4 overexpression induces an accumulation of cells in the G1 phase of the cell cycle (Fig. 2A), suggesting the effects of GPx4 overexpression are largely cytostatic as opposed to cytotoxic. This effect was more pronounced in the U251 cells, likely due to a decreased propensity for contact inhibition over time. Moreover, microscopic analysis of the U251 cells following a 6-day treatment with doxycycline revealed a significant increase in cell surface area from (8613 to 32100 µm^2^, Fig. 2B, C). These results showcase that GPx4 overexpression can block cell cycle progression causing cells to accumulate in G1/G0 phase.

**Fig. 3:**
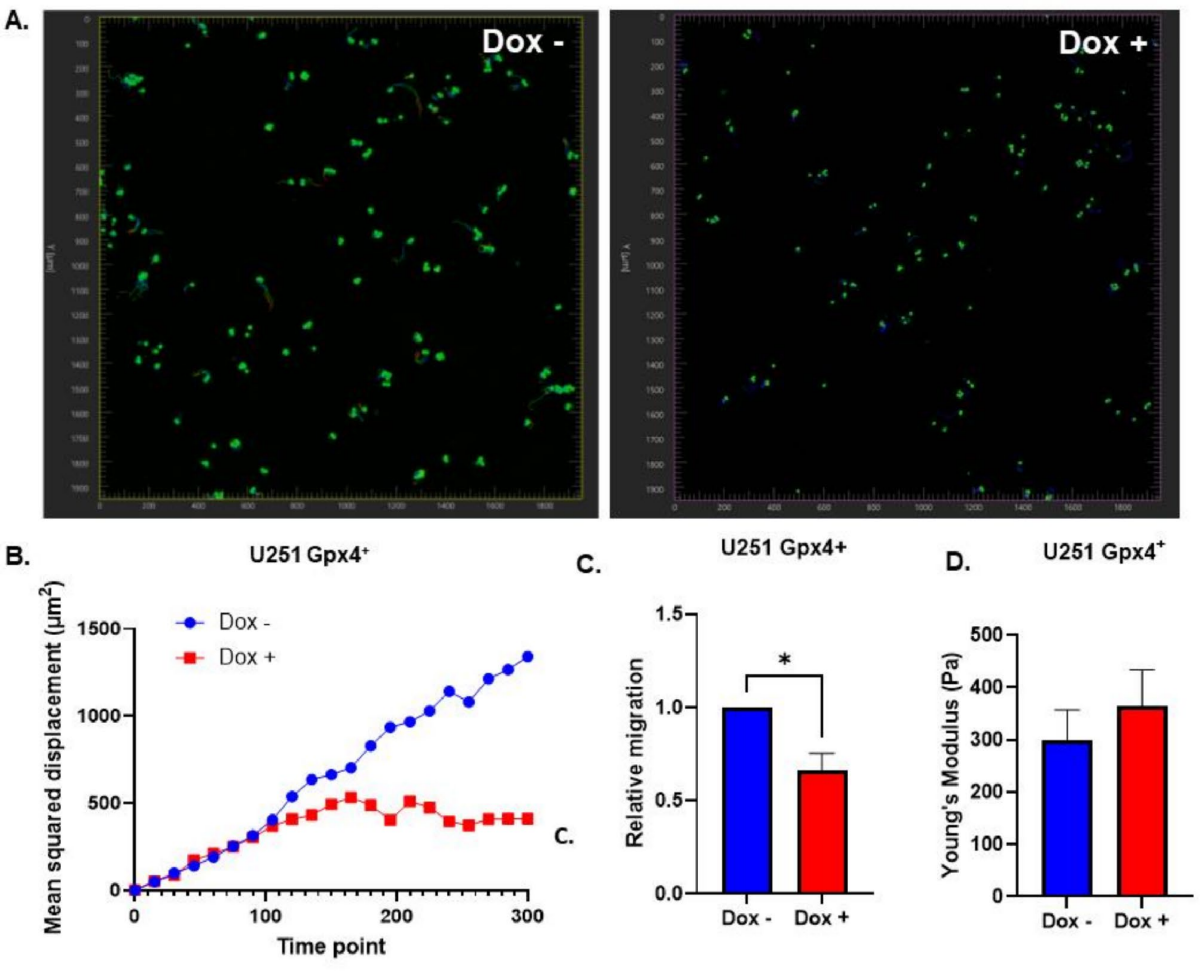
Gpx4 inhibits cell motility and migration. **A/B**: U251 GPx4^+^ cells were treated with 1 µg mL^− 1^ doxycycline for 24 h before re-plating as single cells for cell motility measures. Cells were incubated overnight ± doxycycline prior to the addition of Nucspot 488 live cell stain for confocal imaging. Cells were imaged every 15 min for a total of 300 min to generate cell motility maps using Imaris software (**A**). Imaris software was used to tabulate mean squared displacement for each individual cell (**B**). **C**: Cell migration was analyzed by treating U251 GPx4^+^ cells with 1 µg mL^− 1^ doxycycline for 24 h prior to re-plating on a trans-well membrane for overnight incubation and analysis. Error bars represent mean ± SEM of three independent experiments with *p < 0.05 using a Welch’s T-test. **D**: Cell stiffness was analyzed by treating U251 GPx4^+^ cells with 1 µg mL^− 1^ doxycycline for 24 h prior to re-plating on a glass coverslip. Cells were given an overnight incubation ± doxycycline prior to atomic force microscopic analysis. Error bars represent mean ± SD (*n* = 7–9) with *p < 0.05 using a Welch’s T-test

Based on the long-term morphological changes previously observed in U251 cells, the initial biophysical effects of GPx4 overexpression were investigated. U251 GPx4^+^ cells were treated with 1 µg mL^− 1^ doxycycline for 24 h before re-plating for overnight incubation and biophysical analysis. Live cell motility analysis revealed that U251 cells follow a normal diffusion pattern where mean squared displacement is directly proportional to time elapsed (Fig. 3A, B). However, GPx4 overexpression blunts cell diffusion to promote anomalous sub-diffusion, indicating the inability of cells to take the large random steps associated with the normal random walk motion that can be observed for the control group. Moreover, the overexpression of GPx4 significantly reduces the ability of these cells to migrate (Fig. 3C). Lastly, the changes associated with cell diffusion and invasion led to the interrogation of cell stiffness where it was observed that initial GPx4 overexpression increased U251 cell stiffness from 300 to 365 Pa, however, a high level of heterogeneity prevented statistical significance (*p* = 0.48, Fig. 3D).

**Fig. 4:**
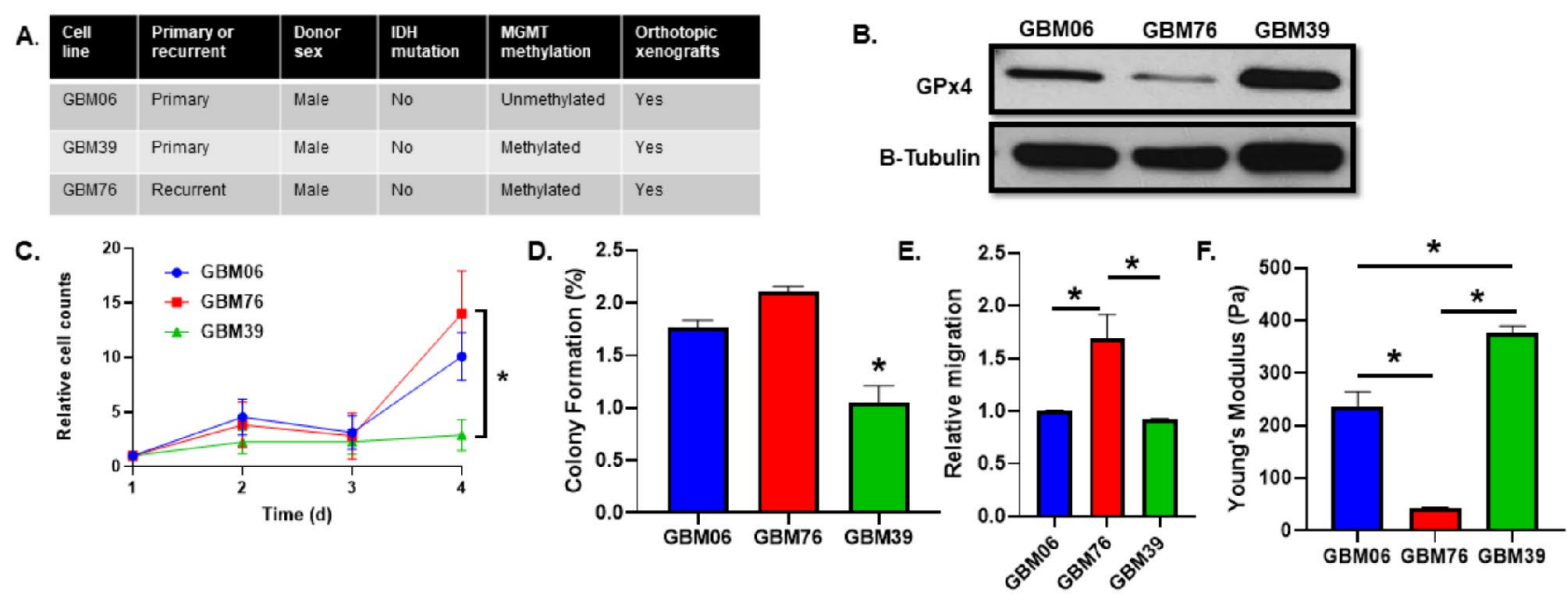
Gpx4 expression correlates with cellular growth and stiffness in patient derived GBM cells. **A**: characteristics of GBM06, 39, and 76 tumor cell lines. **B**: Western blot analysis of GPx4 expression in patient-derived cell lines (GBM06, GBM76, and GBM39). β-tubulin was used as a loading control. **B**: Growth curve of GBM06, GBM76, and GBM39 cell lines. Error bars represent mean ± SD (*n* = 3) with *p < 0.05 using a two-way ANOVA test with post-hoc multiple comparisons to evaluate individual time points. **D/E**: Plating efficiency (**D**) and relative migration (**E**) of GBM06, GBM76, and GBM39 cell lines. Error bars represent mean ± SEM (*n* = 3) with *p < 0.05 using a one-way ANOVA test with a post-hoc Tukey’s test for multiple comparisons. **F**: Cell stiffness analysis of GBM06, GBM76, and GBM39 cell lines using atomic force microscopy. Error bars represent mean ± SD (*n* ≥ 10) with *p < 0.05 using a one-way ANOVA test with a post-hoc Tukey’s test for multiple comparisons

Lastly, this effect was interrogated in patient-derived GBM cells to evaluate the translational relevance of these results. Three separate patient-derived cell lines were evaluated (GBM06, GBM76, and GBM39, Fig. 4A). These cell lines showed a high level of variability in GPx4 expression with GBM39 having the highest and GBM76 having the lowest (Fig. 4B). Interestingly, GBM76 is derived from a recurrent tumor while both GBM06 and GBM39 are derived from primary tumors, consistent with decreased GPx4 expression in recurrent GBM previously described in [9]. Moreover, GBM76 cells grew significantly faster than GBM39 cells but the difference between them and GBM06 did not reach significance (Fig. 4C). Similarly, the colony formation and migration of these patient derived GBM cells were also inversely correlated with GPx4 expression where GBM76 cells showed a significantly greater propensity to form colonies and migrate (Fig. 4D, E). Cell stiffness analysis of these cell lines revealed that GBM76 were also significantly less stiff than the two primary cell lines, where there is an apparent inverse correlation between Young’s modulus and GPx4 expression in these cell lines (Fig. 4F). Therefore, it appears that the inverse relationship between GBM cell growth/stiffness and GPx4 expression is translatable to patient-derived cells in vitro.

## Discussion and conclusions

A recent study evaluating metabolic changes in primary and recurrent GBM tumors revealed significant changes in ferroptosis-related enzymes, with recurrent GBM tumors appearing to exhibit a pro-ferroptosis phenotype [9]. Consistent with these data, a recent study has shown in pre-clinical models that ferroptosis can promote tumor necrosis associated with GBM progression [10]. These data led to the overarching hypothesis that a ferroptosis-prone phenotype supports GBM progression. Moreover, the bioinformatic analysis of the TCGA database revealed similar results where although GPx4 mutations are rare (1.9%), they are most commonly amplifications (5/7), and are associated with significantly greater overall and progression-free survival. Thus, lower levels of GPx4 expression can be inferred to be associated with worse clinical outcomes, although this postulate will require further evaluation. However, to begin to test this hypothesis, we utilized a doxycycline-inducible GPx4 overexpression model system to evaluate the mechanistic impacts of GPx4 overexpression on GBM cell biology.

Importantly, it was observed that overexpressing GPx4 caused a significant decrease in cellular proliferation and colony formation. This was observed in 2 different immortalized cell lines (U251, U118). Moreover, the analysis of patient-cell lines with variable Gpx4 expression revealed an inverse correlation with Gpx4 expression. Further mechanistic analysis revealed that there was a near complete G1 cell cycle arrest in U251 and U118 cells. This was previously corroborated in human breast cancer and liver cancer cells that were genetically engineered to overexpress GPx4 [15, 16]. These results were consistent with changes in cell growth, which were more robust in U251 cells. In conjunction with an accumulation of cells in G1 phase of the cell cycle, there was also a significant increase in cell surface area, suggesting that these cells may be undergoing senescence or entering a quiescent state [17–19].

The robust changes in cell surface area imply a potential biophysical change associated with GPx4 overexpression. At the biophysical level, the overexpression of GPx4 caused an increase in cell stiffness that was unable to reach statistical significance due to the high level of variability in U251 cell stiffness irrespective of GPx4 expression. More convincingly, there was a direct, inverse correlation between cell stiffness and GPx4 expression in patient derived GBM cells. The overexpression of GPx4 increasing cell stiffness remains logical because it has previously been shown that the induction of ferroptosis *via* GPx4 inhibition with RSL3 causes a decrease in cell stiffness [20]. Moreover, a previous study regarding GBM diffusion has reported that stiffness is inversely related to mean squared displacement, however, this work focused primarily on the stiffness of the perivascular space rather than the tumor cells themselves [21]. These data suggest that GBM tumor cell stiffness warrants further consideration as a contributing feature of disease progression.

Despite not observing a significant difference in U251 cell stiffness, the overexpression of GPx4 did significantly impair the diffusion and invasion potential of U251 cells. From a biophysical perspective, U251 cells appear to follow a normal diffusion pattern characteristic of a random walk where the mean squared displacement being proportional to time:

$$\:\langle{\varvec{r}}^{2}\rangle\propto\:\varvec{D}\varvec{\tau\:},$$

where D is the diffusion coefficient and τ is the elapsed time.

However, the overexpression of GPx4 caused U251 cells to undergo anomalous sub-diffusion, indicating that these GBM cells are no longer able to take large, random steps:

$$\:\langle{\varvec{r}}^{2}\rangle\propto\:{\varvec{\tau\:}}^{\varvec{\alpha\:}},\:\varvec{\alpha\:}<1.$$

Therefore, despite observing only moderate changes in cell stiffness, GPx4 overexpression does appear to induce a biophysical change in U251 cells characteristic of impaired growth and motility that warrants further consideration.

Considering recent observations that a pro-ferroptotic phenotype characterizes GBM tumor progression, this study evaluated the biophysical effects associated with GPx4 overexpression. Consistent with the hypothesis that ferroptosis can promote GBM progression, GPx4 overexpression significantly impairs GBM tumor cell growth, plating efficiency, invasion, and diffusion. Overall, it appears that the changes in GBM aggressiveness in vitro are associated with biophysical changes that should be considered in future studies investigating the fundamental mechanisms of GBM progression.

## Electronic supplementary material

Below is the link to the electronic supplementary material.

Supplementary Material 1

## Data Availability

Data is available upon request of the corresponding author.
