# Supplementary material for: Glutathione peroxidase 4 overexpression induces anomalous subdiffusion and impairs glioblastoma cell growth
Source: J Biol Eng. 2024 Dec 21;18:72. doi: 10.1186/s13036-024-00472-x (PMC11663334; doi:10.1186/s13036-024-00472-x)
Supplement: Supplementary file 1 — Supplementary Material 1 [file 13036_2024_472_MOESM1_ESM.docx]

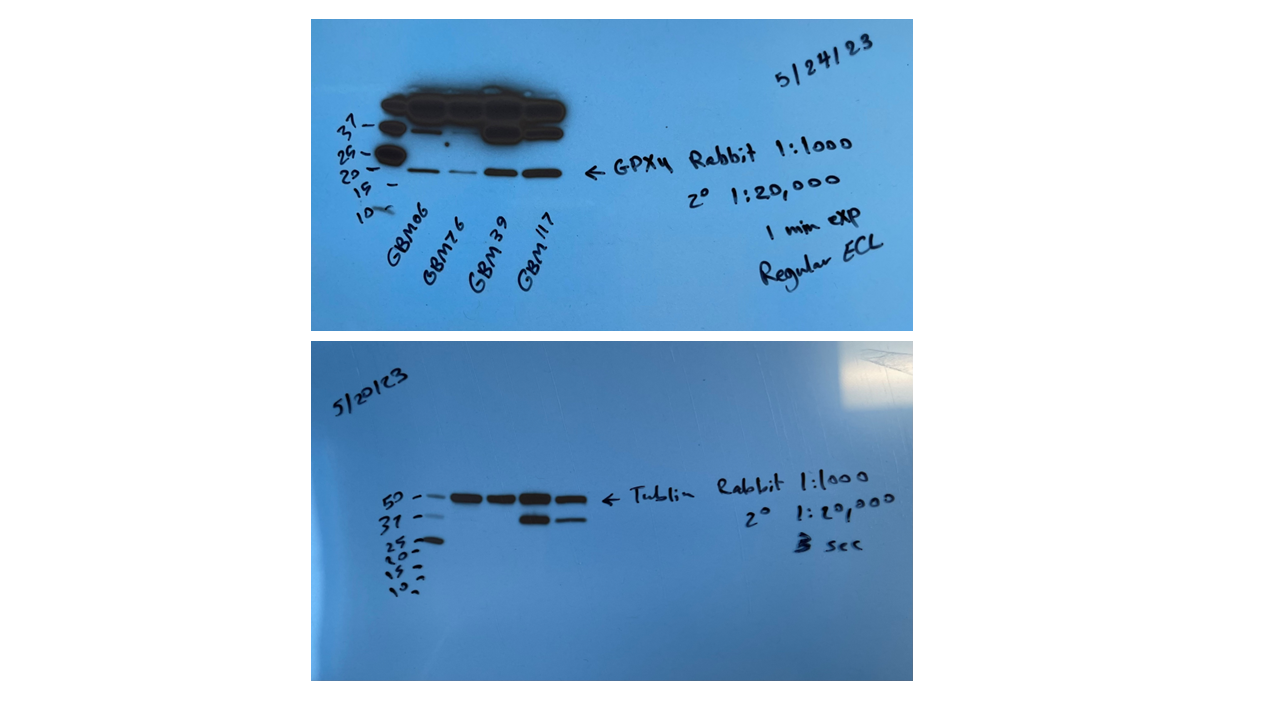


**Unprocessed WBs from Fig. 4:** GPx4 and β-tubulin unprocessed WBs from patient-derived cell lines used in this study.
